# Thirty years of high-voltage injuries: comparative analysis of train surfing and work-related cases with a focus on trauma-associated diagnoses

**DOI:** 10.1007/s00508-025-02591-x

**Published:** 2025-09-02

**Authors:** V. Koenig, A. Fochtmann-Frana, P. Tratnig-Frankl, M. Monai, J. Joestl

**Affiliations:** 1 Division of Plastic, Aesthetic and Reconstructive Surgery, Medical University of Vienna, Waehringer Guertel 18–20, 1090 Vienna, Austria; 2 Private Clinic, Spitalgasse 19, 1090 Vienna, Austria

**Keywords:** High-voltage electrical injuries, Train surfing, Burns, ABSI, ISS, AIS, Glasgow Coma Scale, Injury timing

## Abstract

**Introduction:**

High-voltage electrical injuries from train surfing pose a unique challenge, often involving severe burns and multisystem trauma. This study retrospectively analyzed train surfing injuries over 30 years, with a focus on trauma-associated diagnoses and comparing them to work-related high-voltage injuries.

**Methods:**

A retrospective review of 87 high-voltage injury cases was conducted, including 30 train surfing and 57 work-related cases. Demographics, injury patterns, injury time, intensive care unit (ICU) stays, surgical interventions, Glasgow Coma Scale (GCS) scores, accident timing and trauma scores (Abbreviated Burn Severity Index (ABSI), Injury Severity Score (ISS), Abbreviated Injury Scale (AIS), National Advisory Committee for Aeronautics Score (NACA), Glasgow Outcome Scale (GOS)) were analyzed.

**Results:**

Train surfers were significantly younger (20.6 years vs. 36.0 years, *p* < 0.001) and sustained more severe burns (Total Body Surface Area (TBSA): 46.5% vs. 20.8%, *p* < 0.001). The ABSI (7.1 vs. 5.1, *p* < 0.01) and ISS (25.0 vs. 12.0, *p* < 0.001) were higher in train surfers, reflecting greater injury severity. The ICU stays were longer (56.1 days vs. 15.8 days, *p* < 0.001) and fasciotomies (86.7% vs. 53.0%, *p* < 0.01) and amputations (56.7% vs. 17.5%, *p* < 0.001) were more frequent. Train surfers exhibited worse metabolic parameters (base excess −4.49 vs. −0.7 mmol/L, *p* < 0.001; lactate 3.2 vs. 2.6 mmol/L, *p* < 0.05) and higher AIS scores for head and thoracic trauma. The GCS at the accident site was lower in train surfers (11.1 vs. 13.0, *p* = 0.10), indicating more severe initial impairment. Accidents predominantly occurred at night (69.9% of train surfing cases vs. 3.5% of work-related cases, *p* < 0.001). Mortality was significantly higher in the train surfing group (20.0% vs. 3.2%, *p* < 0.01).

**Conclusion:**

Train surfing injuries involve extensive burns, severe multisystem trauma, and a higher surgical burden compared to work-related injuries. Their distinct injury patterns necessitate targeted prevention and specialized trauma care.

## Introduction

High-voltage electrical injuries (HVEI), particularly from train surfing incidents, pose a unique challenge in polytrauma management [1–3]. Train climbing refers to the act of ascending stationary trains, often driven by curiosity, thrill-seeking or peer influence [4–6]. While these incidents typically occur in train yards or at stations, they pose a severe risk of high-voltage electrocution due to electrical arcing from overhead lines [7–9]. Crucially, even without direct contact, the electrical arc can lead to severe burns and trigger a fall from height, as the affected individuals are often thrown off balance or lose consciousness [4, 10, 11]. As a result, train climbing may also result in significant blunt trauma in addition to electrical injuries. Train surfing, by contrast, involves intentionally riding on top of or between moving train cars. These cases often lead to complex, high-energy trauma patterns combining electrocution, falls at high velocity and collision injuries [2]. These injuries often result in a combination of extensive burns and severe blunt trauma from falls, leading to traumatic brain and spinal cord injuries, thoracic trauma and fractures [10]. Additionally, the electrical current itself causes direct tissue destruction, leading to vascular damage, cardiac arrhythmia and multiorgan failure due to systemic inflammatory response and rhabdomyolysis-induced kidney injury [3]. Unlike occupational high-voltage injuries, train surfing accidents predominantly affect young males and result in complex multisystem trauma requiring aggressive resuscitation and staged surgical interventions [10–13].

Effective polytrauma management follows structured protocols such as Advanced Trauma Life Support (ATLS) and Early Appropriate Care (EAC) [14, 15]. Damage control resuscitation is crucial to prevent hemorrhagic shock, trauma-induced coagulopathy and progressive ischemia [14, 15]. In high-voltage injuries, multiorgan failure is a major concern as the electrical current can cause direct myocardial damage, acute kidney injury from muscle necrosis and respiratory failure due to extensive burns and inhalation injuries [2, 13]. Serial debridement, fasciotomies, and reconstructive strategies are required to manage progressive tissue necrosis, while early critical care interventions aim to prevent secondary organ dysfunction [5, 16].

This study analyzes the intersection of high-voltage trauma and polytrauma, focusing on injury severity, trauma system efficiency, multiorgan failure risk and surgical decision making. Understanding these injury mechanisms is essential for refining trauma care protocols and improving outcomes in this high-risk patient population.

## Material and methods

Following ethical approval (Ethics Committee No. 1384/2023), patient records from January 1994 to December 2024 were reviewed, focusing on high-voltage injuries from train surfing. Data were extracted from the medical records system.

Patients with documented high-voltage injuries were included, while those with low-voltage injuries (< 1000V) or treated outside the department were excluded. Analyzed parameters included age, sex, injury circumstances, TBSA, associated injuries, vitality parameters, ICU stay, polytrauma scores, e.g.,Glasgow Coma Scale (GCS), Glasgow Outcome Scale (GOS), National Advisory Committee for Aeronautics NACA Index, Abbreviated Injury Scale (AIS), Injury Severity Score (ISS) and mortality.

Treatment followed standardized protocols with fluid resuscitation based on urinary output, hematocrit and lactate levels. Myoglobinuria was managed using diuretics and continuous hemodynamic and respiratory monitoring. Current protocols incorporate a tailored approach with balanced crystalloids, albumin, high-dose vitamin C and dynamic monitoring via transpulmonary thermodilution and pulse index contour continuous cardiac output (PiCCO), hemoglobin levels and echocardiography. Circulatory support includes catecholamines (e.g., noradrenaline, dobutamine), alongside ICU care such as oxygen therapy, thermoregulation and analgesia. Initial diagnostics included ENT and ophthalmological consultations, ultrasound, and computed tomography (CT) scans for stabilized patients, with frequent blood tests monitoring creatine kinase and myoglobin.

Decompression surgery (fasciotomies, carpal tunnel release) were performed within 24 h for circular burns or other severe injuries, guided by serum myoglobin and creatine kinase levels. Postoperatively, temporary polyurethane dressings were used, followed by early debridement and amputations when needed. Burn wounds were disinfected and dressed daily, with full-thickness burns treated using split skin grafts or local as well as free flaps for critical defects.

To ensure clarity and comparability, associated injuries in this study were defined as any clinically relevant trauma occurring in addition to high-voltage burns and directly attributable to the injury mechanism. These injuries were categorized into three groups:

1. Neurological injuries, including intracranial bleeding (ICB), skull fractures, and spinal cord injuries.
2. Thoracic injuries, encompassing pneumothorax and rib fractures.
3. Orthopedic injuries, such as spinal fractures (without cord involvement), long bone fractures, and clavicle fractures.

## Statistical analysis

Data analysis was conducted using R Version 3.1.1 and SPSS (IBM, Armonk, NY, USA). Student’s t‑test was used for parametric data, and the Mann–Whitney U test for non-parametric data. Categorical variables were assessed via the χ^2^-testor Fisher’s exact test. Correlations between the groups were analyzed using contingency tables and χ^2^-tests. Statistical significance was set at *p* < 0.05. Parametric data are presented as mean ± standard deviation, while nonparametric data are shown as median (min–max).

## Results

A total of 93 cases were initially identified, with 6 cases excluded due to incomplete prehospital records and missing data, resulting in 87 cases included in the analysis. The mean age of all high voltage electrical injury (HVEI) patients was 31.0 years (range: 13–73 years), with a strong male predominance of 96.8% (*p* < 0.0001). The mean total body surface area (TBSA) affected was 29.1% (range: 1–80%). The mean Abbreviated Burn Severity Index (ABSI) was 5.7 (range: 3.0–12.0), while the mean Injury Severity Score (ISS) was 16.4 (range: 4.0–57.0). The mean intensive care unit (ICU) stay was 28.8 days (range: 1–270 days). The overall mortality rate was 8.6%, with an incidence of cardiac arrest in 11.8% and neurological findings in 27.2% of cases. The causes of death in this cohort were multifactorial, with the predominant contributors being multiorgan failure secondary to extensive burn injury, prolonged ICU stay, acute kidney failure and septic complications arising from wound infections. These outcomes reflect the systemic burden of high-voltage trauma, particularly in train surfing patients with extensive TBSA involvement, necessitating complex surgical care, repeated debridement and prolonged mechanical ventilation, all of which increase susceptibility to infection and progressive organ dysfunction. All recorded deaths occurred during the intensive care unit stay. Associated injuries were present in 41.9% of patients.

Of the 63 work-related cases, s6ix were excluded due to incomplete emergency response documentation and missing clinical data, leaving 57 cases for analysis. A total of 30 cases were identified in the train surfer injury subgroup. The mean age was significantly lower at 20.6 years (range: 13–52 years) compared to 36.0 years (range: 18–73 years) in the work-related group (*p* < 0.001). Males comprised 93.3% of cases in the train surfer group versus 98.4% in the occupational cohort. The mean TBSA of train surfers was markedly higher at 46.5% (range: 12–80%), significantly exceeding the 20.8% (range: 1–70%) observed in occupational injuries (*p* < 0.001). Similarly, the mean ABSI score was elevated at 7.1 (range: 3.0–12.0), compared to 5.1 (range: 3.0–11.0) in the work-related group (*p* < 0.001). Injury severity, as reflected by the ISS score, was also significantly higher among train surfers (25.0 vs. 12.0; *p* < 0.001) (Table 1).

**Table 1:** Summary of the train surfing and work-related injuries included in this study

Parameter	Train surfing Injuries	Work-related Injuries	*p*-value
Number of cases	30.0	57.0	–
Mean age (years)	20.6	36.0	< 0.001
Male (%)	93.3	98.4	n. s.
Mean TBSA (%)	46.5	20.8	< 0.001
Mean ABSI	7.1	5.1	< 0.001
Mean ICU stay (days)	56.1	15.8	< 0.001
Mortality rate (%)	20.0	3.2	18
Cardiac arrest (%)	20.0	7.9	0.166
Mean GCS	11.1	13.0	n. s.
Mean ISS	25.0	12.0	< 0.001
Mean GOS	4.03	4.56	n. s.
AIS head	1.57	0.37	< 0.001
AIS thorax	1.17	0.14	1
AIS external	3.9	3.26	3
Neurological findings (%)	51.7	15.9	1
Associated injuries (%)	73.3	27.0	< 0.001
Spinal fractures (*n*)	7.0	2.0	7
Spinal cord injuries (*n*)	4.0	1.0	26
Skull fractures (*n*)	11.0	1.0	< 0.001
Intracranial bleeding (*n*)	8.0	1.0	1
Pneumothorax (*n*)	9.0	4.0	46
Mean systolic BP (mm Hg)	118.78	137.87	16
Mean diastolic BP (mm Hg)	65.96	75.91	6
Base excess	−4.49	−0.7	1
Lactate (mmol/L)	3.2	2.6	34

*ABSI* abbreviated burn severity index, *AIS* Abbreviated Injury Scale, *BP* blood pressure, *GCS* Glasgow Coma Scale, *GOS* Glasgow Outcome Scale, *ICU* intensive care unit, *ISS* Injury Severity Score, *TBSA* total body surface area

**Fig. 1:**
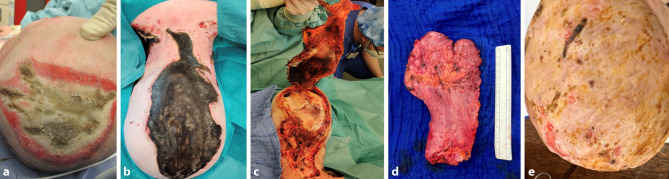
Clinical course of a high-voltage electrical injury (HVEI) with arc entry point at the scalp. Third-degree burns with full-thickness tissue necrosis required debridement, defect coverage was achieved using a free latissimus dorsi flap and split-thickness skin grafting. Photographic documentation of the electrical entry site on the head at admission, prior to debridement, and intraoperatively

**Fig. 2:**
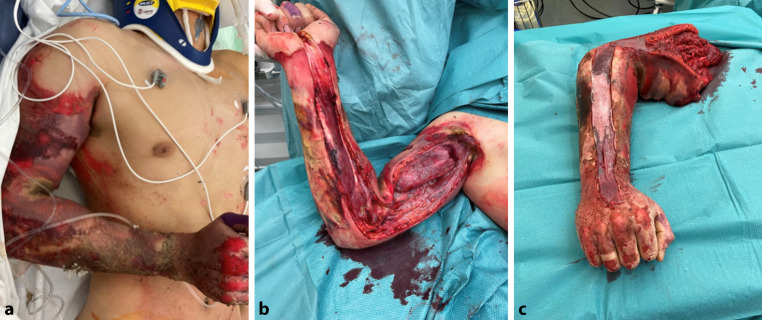
This patient sustained a high-voltage electrical injury after holding a metallic object in his right hand, which served as the entry point for the arc. Due to full-thickness (third-degree) burns, the upper extremity required amputation. The resulting defect was reconstructed using a pedicled latissimus dorsi flap. The patient is currently scheduled for osseointegration in preparation for a bionic hand prosthesis [29]

The distribution of entry and exit sites reflected the typical current flow patterns observed in high-voltage electrical injuries published before. In train surfing cases, vertical current flow was predominant, typically originating at the head or upper extremities and exiting through the lower limbs (Fig. 1). This vertical trajectory increases the likelihood of transthoracic and transabdominal current passage, placing vital organs such as the heart, lungs, and kidneys at considerable risk. In contrast, work-related injuries more frequently exhibited diagonal current flow, with entry often occurring through the upper extremities, usually the dominant and workinghand and exiting at the lower extremity. This pattern is characteristic of occupational contact with energized equipment or tools (Fig. 2).

The ICU stay was prolonged in the train surfing group, with a mean of 56.1 days (range: 1–270 days), significantly longer than the 15.8 days (range: 1–111 days) seen in work-related injuries (*p* < 0.001). The mortality rate was significantly higher at 20.0% versus 3.2% in the occupational group (*p* = 0.018). Cardiac arrest occurred in 20.0% of train surfers versus 7.9% in work-related cases, although this difference did not reach statistical significance (*p* = 0.166). Neurological findings were observed in 51.7% of train surfers compared to 15.9% in the reference group (*p* = 0.001), and associated injuries were significantly more frequent among train surfers (73.3% vs. 27.0%; *p* < 0.001).

When analyzing the time of injury occurrence, 24.1% of all cases (*n* = 21) were documented between 09:00–11:59 and another 24.1% (*n* = 21) between 12:00–14:59. The least frequent time interval was 06:00–08:59, accounting for only 2.3% of all cases (*n* = 2).

Train surfer injuries most commonly occurred during nighttime hours, with the highest incidence recorded between 00:00–02:59 and 03:00–05:59, each accounting for 23.3% of train surfing cases (*n* = 7, respectively). A substantial proportion also occurred between 21:00–23:59 (20.0%, *n* = 6). No train surfer injuries were reported during 06:00–08:59 or 12:00–14:59.

In contrast, work-related injuries occurred almost exclusively during standard working hours. The peak frequencies were observed between 09:00–11:59 (33.3%, *n* = 19) and 12:00–14:59 (36.8%, *n* = 21), with a minor proportion between 06:00–08:59 (3.5%, *n* = 2). Notably, no work-related injuries were recorded during 00:00–05:59.

These differences in injury timing patterns between the two groups were statistically significant (*p* < 0.001), reflecting the influence of behavioral context and occupational activity cycles on the timing of high-voltage trauma (Table 2).

**Table 2:** Time interval of accidents

Time interval	Total cases (*n*)	Total cases (%)	Train surfer cases (*n*)	Train surfer cases (%)	Work-related cases (*n*)	Work-related cases (%)
00:00–02:59	7	8.0	7	23.3	0	0.0
03:00–05:59	7	8.0	7	23.3	0	0.0
06:00–08:59	2	2.3	0	0.0	2	3.5
09:00–11:59	21	24.1	2	6.7	19	33.3
12:00–14:59	21	24.1	0	0.0	21	36.8
15:00–17:59	15	17.2	5	16.7	10	17.5
18:00–20:59	4	4.6	2	6.7	2	3.5
21:00–23:59	9	10.3	6	20.0	3	5.3

Train surfers had significantly lower Glasgow Coma Scale (GCS) scores at the accident site compared to work-related injuries (11.1 vs. 13), indicating more severe initial impairment, although not statistically significant. The NACA index was significantly higher in train surfers, reflecting greater injury severity (4.7 vs. 3.8, *p* = 0.04). Intubation was required in 56.7% of train surfer cases compared to 29.8% of work-related cases, further emphasizing the severity of injuries in the former group. Resuscitation rates were comparable between the two cohorts (6.7% vs. 7.0%).

**Fig. 3:**
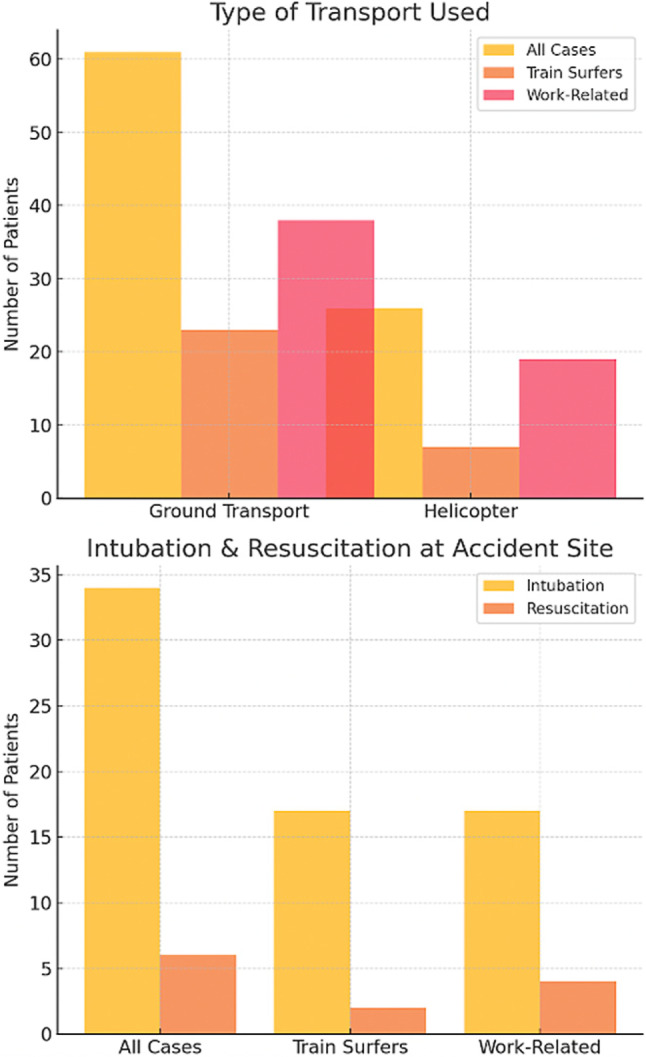
Overview of transport modalities to the hospital, as well as intubation and resuscitation at the accident site

A total of 87 patients were transported, with 61 arriving by emergency ambulance and 26 by helicopter. Among the 30 train surfers, 22 were transported by ambulance and 8 by helicopter. In work-related accidents, 38 patients were transported by ground ambulance, while 19 required helicopter transport. Helicopter transport was used more frequently in work-related cases (33.3% vs. 23.3%), possibly due to the remoteness of workplaces and the timing of the accidents (Fig. 3).

**Fig. 4:**
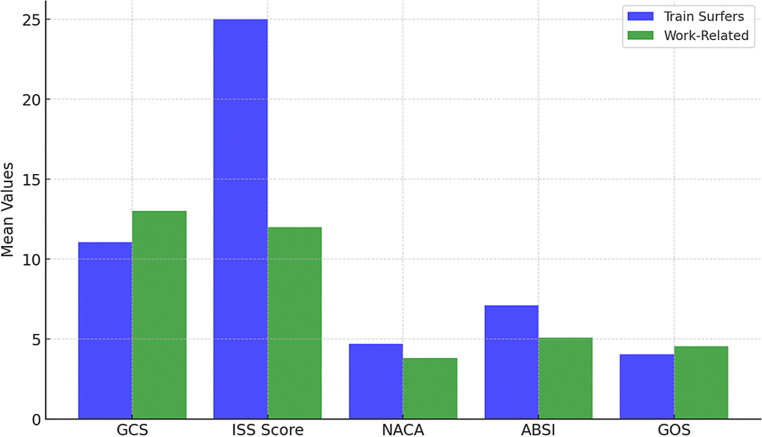
Comparison of GCS, ISS, NACA, ABSI and GOS scores for train surfers and work-related with high-voltage injuries

Functional outcomes were also worse in train surfers, as reflected in the Glasgow Outcome Scale (GOS), where train surfers had a mean score of 4.03 compared to 4.56 in work-related injuries. The difference in mean GOS suggests that train surfers faced poorer long-term recovery prospects. The overall data underscore the significantly worse prognosis associated with train surfing injuries and highlight the necessity of targeted prevention strategies (Fig. 4).

Train surfers exhibited significantly different vital parameters compared to work-related accident cases. Their mean heart rate was higher (98.96 bpm, range: 46–129) compared to work-related injuries (92.06 bpm, range: 61–153), potentially indicating heightened physiological stress or a more pronounced systemic response to injury. Respiratory frequency was also elevated in train surfers (17.17 breaths/min, range: 9–43) versus work-related cases (15.57 breaths/min, range: 10–21), further supporting the notion of increased respiratory demand.

Blood pressure measurements revealed a trend toward lower systolic and diastolic values in train surfers. Mean systolic blood pressure was 118.78 mm Hg (range: 80–180) compared to 137.87 mm Hg (range: 80–210) in work-related injuries (*p* = 0.016), while diastolic blood pressure was also lower (65.96 mm Hg, range: 42–120) versus 75.91 mm Hg (range: 41–110) in the occupational group (*p* = 0.006). This could suggest a higher prevalence of hypovolemia or different injury mechanisms in train surfing accidents.

Metabolic parameters further reinforced the severity of physiological disturbances in train surfers. Base excess was significantly more negative (−4.49, range: −17.0 to 2.5) compared to work-related injuries (−0.7, range: −6.3 to 5.6) (*p* = 0.001), indicating a greater degree of metabolic acidosis, which is often associated with severe trauma, shock, or prolonged hypoxia. Additionally, lactate levels were higher (3.2 mmol/L, range: 1.0–7.8) compared to work-related cases (2.6 mmol/L, range: 0.7–6.1) (*p* = 0.034), suggesting an increased anerobic metabolism due to tissue hypoxia.

**Fig. 5:**
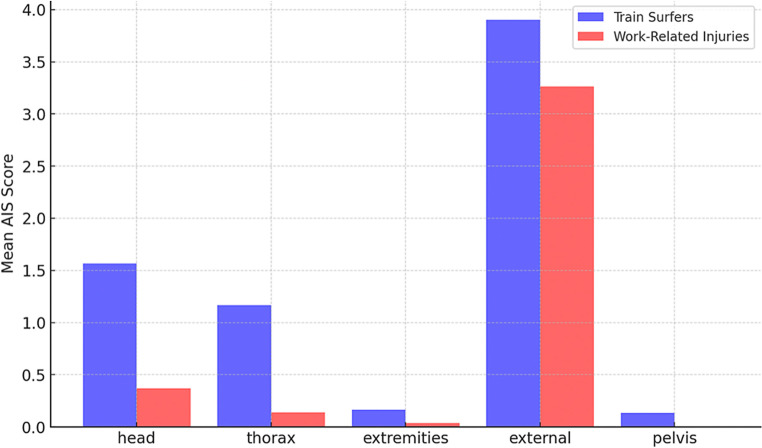
Mean AIS scores by body region

Injury severity, as measured by the Abbreviated Injury Scale (AIS), revealed significant differences in affected body regions. Train surfers exhibited higher AIS scores for head injuries (mean: 1.57, range: 0–5) compared to work-related injuries (mean: 0.37, range: 0–3) (*p* < 0.001), suggesting a greater impact on neurological function. Thoracic injuries were also more severe in train surfers (mean: 1.17, range: 0–5) than in work-related cases (mean: 0.14, range: 0–3) (*p* = 0.001), possibly indicating high-energy trauma mechanisms. External injuries were particularly pronounced in train surfers (mean: 3.90, range: 2–5) compared to work-related cases (mean: 3.26, range: 2–5) (*p* = 0.003), highlighting the extensive nature of surface trauma in this cohort. These findings further emphasize the unique injury patterns seen in train surfers and reinforce the need for targeted prevention and treatment strategies (Fig. 5).

Train-surfing cases were associated with a significantly higher frequency of all three major injury categories, neurological (e.g., intracranial hemorrhage, spinal cord injury), thoracic (e.g., pneumothorax, rib fractures) and orthopedic injuries (e.g., spinal and skull fractures, extremity trauma), with neurological injuries being particularly prominent in this subgroup (*p* < 0.001) (Table 3).

**Table 3:** Injury categories

Injury category	Train surfing cases	Work-related cases	*p*-value
Neurological (ICB, spinal cord injury, skull fracture)	23	4	< 0.001
Thoracic (pneumothorax, rib fracture)	12	4	0.048
Orthopedic (spinal fracture, long bone, clavicle)	10	2	0.039

*ICB* xxxx

A total of 9 spinal fractures were documented, 7 of which occurred in the train surfing group and 2 in work-related cases (*p* = 0.007); 5 of these resulted in spinal cord injuries, with 4 cases in the train surfing subgroup (*p* = 0.026). Surgical intervention was performed in 4 cases, while the remaining fractures were managed conservatively, either due to the less severe nature of the injury or because the patients were enrolled in an optimal handling protocol for severe head trauma, which precluded early fracture stabilization. Additionally, 12 skull fractures were recorded, predominantly among train surfers (11 cases) compared to a single work-related case (*p* < 0.001). Intracranial hemorrhages were observed in 9 patients, with 8 occurring in train surfers and 1 in a work-related injury (*p* = 0.001). These included 3 subdural hematomas, 7 epidural hematomas, and 5 cases of subarachnoid bleeding, with some patients exhibiting multiple types of intracranial hemorrhage. Thoracic injuries comprised 13 cases of pneumothorax, with 9 occurring in train surfers (*p* = 0.046), and 5 rib fractures, of which 3 were found in train surfers. Fractures of the extremities and clavicle were noted in isolated cases and were not significantly different between groups.

### Limitations

This study is limited by its retrospective design, 6 patients were excluded due to missing prehospital data, reducing the final cohort to 87 cases. Accurate assessment of time from injury to admission was not possible, particularly in train surfers, who were often found alone and at night, leading to assumed delays in rescue.

## Discussion

This study provides a comprehensive retrospective analysis of high-voltage electrical injuries (HVEI), with a focus on the particularly severe trauma patterns associated with train surfing incidents. Our findings are consistent with and expand upon existing literature by highlighting the disproportionately high morbidity and mortality among this young, high-risk subgroup [1, 5, 12, 16–20].

Korkiamäki et al. reported a median TBSA of 45% and a 16.7% in-hospital mortality in 18 train climbers over a 30-year period in Helsinki, mirroring our findings of a mean TBSA of 46.5% and a 20.0% mortality rate among train surfers [1]. Our data further show a significantly prolonged ICU stay (mean: 56.1 days), a high incidence of associated injuries (73.3%), and neurological impairment (51.7%), emphasizing the systemic burden of injuries sustained through this mechanism. These results align with the outcomes described by Sternick et al. and Koller et al., who also documented devastating injuries resulting from contact with railway overhead cables, often caused by electrical arcs without direct contact [21, 22]. The pattern of injuries we observed supports the hypothesis that arc-induced burns and falls from elevated positions both contribute to the severity of train surfing trauma.

Our results also closely reflect those of Gille et al., who analyzed 162 electrical burn patients and found that high-voltage injuries were significantly associated with cardiac arrest, loss of consciousness, and amputation [3]. Similarly, we noted an 11.8% incidence of cardiac arrest in our overall cohort, with significantly worse metabolic parameters and base excess levels among train surfers, indicating more pronounced shock states. Dash et al. identified a 38% amputation rate in electrical burn injuries, particularly in high-voltage and contact burns, further supporting the notion that high-voltage trauma, particularly when combined with falls, results in extensive systemic and musculoskeletal damage [23].

Hussmann et al. highlighted a high rate of major limb amputation (35%) and long-term neurological deficits (73%) in high-voltage injuries, primarily in work-related settings [13]. While our study did not focus on amputations, the neurological impairment rate of 51.7% and TBSA values far exceeding those reported by Hussmann et al. (9.5%) indicate a greater initial severity in train surfers. This discrepancy may be attributed to the unique injury mechanism, where electrical trauma is frequently coupled with high-energy falls, leading to more severe polytrauma and systemic compromise. Furthermore, our data revealed worse Glasgow Outcome Scores and higher ICU treatment burden, reinforcing the profound functional and clinical implications of train surfing injuries.

Cancio et al. emphasized the frequent development of compartment syndrome and amputations in high-voltage injuries, reporting a 41% amputation rate [10]. Although our study did not specifically quantify these interventions, the extensive external trauma (mean AIS score of 3.90), high TBSA, and frequent neurological and head injuries in our train surfer subgroup suggest similarly severe systemic impacts. Notably, while Cancio et al. reported a 4.1% mortality rate, we found a 20.0% rate among train surfers, likely reflecting the added trauma burden from falls, complex polytrauma and delays in rescue due to the nature and timing of such accidents.

Berwin et al. discussed shifts in polytrauma care strategies, advocating physiology-guided early appropriate care (EAC) [15]. Our findings of significantly prolonged ICU stay in train surfers support the notion that conventional protocols may not be adequate in this population. Train surfers may require highly individualized approaches integrating trauma, burn, cardiac and neurocritical care pathways. The systemic physiological derangements we observed, such as elevated lactate, negative base excess, and hypotension, suggest that early physiological monitoring could aid in risk stratification and inform early interventions.

Martinez Chamorro et al. underscored the importance of early whole-body CT imaging in polytrauma [24]. In our study, frequent findings, such as spinal fractures, skull fractures, and thoracic injuries (e.g., pneumothorax in 30% of train surfers) support this recommendation. Rapid imaging is crucial for the timely detection of life-threatening complications in train surfing injuries, where the combination of blunt and electrical trauma can obscure clinical presentation.

While the observed difference in Glasgow Coma Scale (GCS) scores between train surfers and work-related injury patients did not reach statistical significance (mean GCS 11.1 vs. 13.0; *p* = 0.10), this trend remains clinically noteworthy. The GCS is a routinely used parameter in trauma assessment and, although not a novel predictor, it remains a valuable tool for estimating injury severity and the need for early intervention. In our cohort, the lower GCS in train surfers is consistent with the more frequent occurrence of severe head trauma, spinal injuries, and associated neurological findings in this subgroup. As highlighted by Vorbeck et al., GCS continues to be a relevant early marker of morbidity and mortality risk in polytrauma patients [25]. Although not statistically conclusive in our data, the trend reflects the overall clinical picture of greater injury burden in train surfing incidents and should be considered in the context of prehospital triage and early critical care decision making. The studies of Marsden and Vorbeck et al. identified early predictors of mortality in polytrauma patients, including low Glasgow Coma Scale (GCS), cardiopulmonary resuscitation (CPR), and base excess [14, 24, 26]. Our data support these associations: train surfers had significantly lower GCS scores, higher rates of intubation, more frequent CPR, and worse metabolic parameters. These findings reinforce the need for rapid triage tools and emphasize that train surfing injuries represent a high-mortality subgroup, even within the context of high-voltage trauma.

Schroeter et al. showed that prehospital ISS is often underestimated in patients with ISS ≥ 25 [26]. In our study, the mean ISS in train surfers was 25.0, and severe head and thoracic injuries were common. This supports the concern that train surfing injuries may be initially underappreciated in severity, underscoring the need for enhanced prehospital protocols and better early recognition of this injury mechanism.

In our cohort, all recorded fatalities occurred during intensive care unit (ICU) treatment and were the result of multifactorial complications [7]. The leading causes of death included multiorgan failure secondary to extensive burn injuries, acute kidney failure, and septic complications arising from wound infections. Notably, prolonged ICU stays, repeated surgical debridement, and extended periods of mechanical ventilation contributed to systemic deterioration, particularly in train surfing patients with extensive TBSA involvement. These findings align with those previously described by Koenig et al., who emphasized the compounding effects of high-voltage trauma and polytrauma, especially in cases involving vertical current flow and additional injuries from falls, on the risk of organ failure and fatal outcomes [28]. Their work also highlighted the significant role of secondary injuries and delayed resuscitation in shaping clinical trajectories using a multivariate logistic regression to identify three independent predictors of mortality: total body surface area burned (TBSA), the need for cardiopulmonary resuscitation and the presence of acute kidney failure. These findings emphasize the importance of early identification of life-threatening systemic complications.

Taken together, these results illustrate the substantial mortality burden associated with high-voltage injuries, particularly in train surfers and reinforce the need for individualized risk assessment, aggressive early resuscitation and close monitoring for secondary organ failure. They also suggest that fatal outcomes in this cohort are not solely a function of the electrical insult itself but more the result of a cascading interplay between burn severity, physiological shock, renal impairment and delayed critical care, especially in the context of underestimated prehospital presentations.

Finally, Koenig et al. documented the devastating impact of train surfing trauma in a young Austrian population and initiated an awareness campaign to address this public health concern [7]. Our findings confirm the extreme severity of these injuries and add further evidence supporting targeted prevention efforts. Moreover, our study highlights that many train surfers likely sustained injuries via electrical arcs and secondary falls rather than direct contact, as Koller et al. also suggested. This arc-induced trauma leads to ignition of clothing and rapid incapacitation, often resulting in severe burns and falls from height.

In conclusion, our study confirms that train surfing-related high-voltage injuries represent a distinct and highly lethal trauma pattern [12, 16, 27]. Compared to conventional work-related high-voltage injuries, these cases involve younger patients, greater burn extent, more frequent neurological involvement and significantly longer ICU stays. The literature corroborates these observations and emphasizes the urgent need for prevention strategies, early imaging, physiological monitoring and individualized multidisciplinary care. Future research should explore targeted intervention pathways and evaluate the long-term outcomes of survivors in this particularly vulnerable population.

## Conclusion

The data highlight significant differences between train surfer and work-related injuries. Train surfer injuries predominantly affect younger males and result in more severe injuries, with higher TBSA involvement, longer ICU stays, and greater associated mortality. These injuries commonly occur during nighttime hours. In contrast, work-related injuries are more prevalent in older individuals, with lower severity scores, shorter ICU stays, and lower mortality rates, occurring primarily during daytime working hours. The distinct injury patterns and time distributions emphasize the need for targeted prevention strategies for these two groups.

Overall, the comparison highlights that train surfers experience greater physiological stress, metabolic derangement, and potential hemodynamic instability compared to work-related trauma cases. These findings emphasize the need for aggressive early intervention and monitoring in train surfing injuries.

## References

[1] Korkiamaki A, et al. Electrical burns in train climbers treated in the helsinki burn centre during the last 30 years. Scand J Trauma Resusc Emerg Med. 2024;32(1):112. 39533295 10.1186/s13049-024-01283-1 PMC11556043

[2] Khor D, et al. Electrical injuries and outcomes: a retrospective review. Burns. 2023;49(7):1739–44. 37005139 10.1016/j.burns.2023.03.015

[3] Gille J, et al. Electrical injury—a dual center analysis of patient characteristics, therapeutic specifics and outcome predictors. Scand J Trauma Resusc Emerg Med. 2018;26(1):43. 29855384 10.1186/s13049-018-0513-2 PMC5984367

[4] Cuculic D, Sosa I. “Selfie”-related electrocution. Forensic Sci Med Pathol. 2019;15(4):635–7. 30649689 10.1007/s12024-018-0078-4

[5] Lumenta DB, et al. Train surfing and other high voltage trauma: differences in injury-related mechanisms and operative outcomes after fasciotomy, amputation and soft-tissue coverage. Burns. 2011;37(8):1427–34. 21852047 10.1016/j.burns.2011.07.016

[6] Strauch H, Wirth I, Geserick G. Fatal accidents due to train surfing in Berlin. Forensic Sci Int. 1998;94(1-2):119–27. 9670490 10.1016/s0379-0738(98)00064-4

[7] Koenig V, et al. Train climbing—A new old trend in adolescents: treatment of high voltage injuries and planning of a pilot project to raise awareness. Wien Klin Wochenschr. 2024;136(19-20):570–4. 39172198 10.1007/s00508-024-02399-1 PMC11464593

[8] Arnoldo BD, et al. Electrical injuries: a 20-year review. J Burn Care Rehabil. 2004;25(6):479–84. 15534455 10.1097/01.bcr.0000144536.22284.5c

[9] Butler ED, Gant TD. Electrical injuries, with special reference to the upper extremities. A review of 182 cases. Am J Surg. 1977;134(1):95–101. 879415 10.1016/0002-9610(77)90290-2

[10] Cancio LC, et al. One hundred ninety-five cases of high-voltage electric injury. J Burn Care Rehabil. 2005;26(4):331–40. 16006840 10.1097/01.bcr.0000169893.25351.a9

[11] Ferreiro I, et al. Factors influencing the sequelae of high tension electrical injuries. Burns. 1998;24(7):649–53. 9882065 10.1016/s0305-4179(98)00082-5

[12] Maghsoudi H, Adyani Y, Ahmadian N. Electrical and lightning injuries. J Burn Care Res. 2007;28(2):255–61. 17351442 10.1097/BCR.0B013E318031A11C

[13] Hussmann J, et al. Electrical injuries—morbidity, outcome and treatment rationale. Burns. 1995;21(7):530–5. 8540982 10.1016/0305-4179(95)00037-c

[14] Marsden NJ, Tuma F. Polytraumatized patient, in statpearls. Treasure Island (FL); 2025. 32119313

[15] Berwin JT, et al. Managing polytrauma patients. Injury. 2020;51(10):2091–6. 32758368 10.1016/j.injury.2020.07.051

[16] Shih JG, Shahrokhi S, Jeschke MG. Review of adult electrical burn injury outcomes worldwide: an analysis of low-voltage vs high-voltage electrical injury. J Burn Care Res. 2017;38(1):e293–e8. 27359191 10.1097/BCR.0000000000000373 PMC5179293

[17] Seyfrydova M, et al. Arrhythmias and laboratory abnormalities after an electrical accident: a single-center, retrospective study of 333 cases. Clin Res Cardiol. 2023;112(12):1835–47. 37526697 10.1007/s00392-023-02274-5

[18] Sanford A, Gamelli RL. Lightning and thermal injuries. Handb Clin Neurol. 2014;120:981–6. 24365365 10.1016/B978-0-7020-4087-0.00065-6

[19] Kym D, et al. Epidemiology of electrical injury: differences between low- and high-voltage electrical injuries during a 7-year study period in south korea. Scand J Surg. 2015;104(2):108–14. 24809357 10.1177/1457496914534209

[20] Luce EA, Gottlieb SE. “true” high-tension electrical injuries. Ann Plast Surg. 1984;12(4):321–6. 6721393 10.1097/00000637-198404000-00003

[21] Koller J. High-tension electrical-arc-induced thermal burns caused by railway overhead cables. Burns. 1991;17(5):411–4. 1760113 10.1016/s0305-4179(05)80077-4

[22] Sternick I, et al. “train surfers”: analysis of 23 cases of electrical burns caused by high tension railway overhead cables. Burns. 2000;26(5):470–3. 10812270 10.1016/s0305-4179(99)00173-4

[23] Dash S, et al. Study of clinical pattern of limb loss in electrical burn injuries. Injury. 2021;52(7):1925–33. 33902868 10.1016/j.injury.2021.04.028

[24] Martinez Chamorro E, et al. Patients with severe polytrauma: management and imaging protocols. Radiologia. 2023;65(1):S11–S20. 37024226 10.1016/j.rxeng.2022.09.008

[25] Vorbeck J, et al. Mortality risk factors of severely injured polytrauma patients (prehospital mortality prediction score). J Clin Med. 2023;12(14). 10.3390/jcm12144724 PMC10380896 37510839

[26] Schroter C, et al. Injury severity in polytrauma patients is underestimated using the injury severity score: a single-center correlation study in air rescue. Eur J Trauma Emerg Surg. 2019;45(1):83–9. 29234837 10.1007/s00068-017-0888-1

[27] Lumenta DB, Kamolz LP, Frey M. Adult burn patients with more than 60 % TBSA involved-meek and other techniques to overcome restricted skin harvest availability—the viennese concept. J Burn Care Res. 2009;30(2):231–42. 19165111 10.1097/BCR.0b013e318198a2d6

[28] Koenig V, Joestl J, Ihra G, Windpassinger M, Monai M, Fochtmann-Frana A. Thirty years of experience with high-voltage injuries: mechanisms, current flow patterns, and implications for cardiac and renal failure in train-surfing vs. Work-related cases. J Clin Med. 2025;14:2659. 10.3390/jcm14082659. 40283489 10.3390/jcm14082659 PMC12028272

[29] Sturma A, et al. Long-term functional and clinical outcome of combined targeted muscle reinnervation and osseointegration for functional bionic reconstruction in transhumeral amputees: a case series. J Rehabil Med. 2024;56:jrm34141. 38770700 10.2340/jrm.v56.34141 PMC11135336

